# Can invasive interventions be avoided with a holistic swallowing therapy program in older patients in intensive care units: percutaneous endoscopic gastrostomy tubes or oral intake?

**DOI:** 10.3389/fneur.2025.1484493

**Published:** 2025-01-22

**Authors:** Çağla Eliküçük, Fatma Esen Aydinli, Nazan Has Selmi, Cansu Uzunoğlu, Murathan Köksal, Volkan Öter, Belgin Akan, Erdal Birol Bostanci, Güldeniz Argun

**Affiliations:** ^1^Speech and Language Therapy Clinic, Ankara Bilkent City Hospital, Ankara, Türkiye; ^2^Department of Speech and Language Therapy, Faculty of Health Sciences, Hacettepe University, Ankara, Türkiye; ^3^Intensive Care Unit, Ankara Bilkent City Hospital, Ankara, Türkiye; ^4^Neurology Intensive Care Unit, Ankara Bilkent City Hospital, Ankara, Türkiye; ^5^Radiology Clinic, Ankara Bilkent City Hospital, Ankara, Türkiye; ^6^Gastroenterology Surgery Service, Ankara Bilkent City Hospital, Ankara, Türkiye; ^7^Intensive Care Unit, Ankara Bilkent City Hospital, Ankara, Türkiye; ^8^Anesthesiology and Reanimation, Yenimahalle, Ankara Oncology Training and Research Hospital, Ankara, Türkiye

**Keywords:** geriatrics, dysphagia, nutrition, therapeutics, intensive care units

## Abstract

**Introduction:**

The use of percutaneous endoscopic gastrostomy (PEG) tubes in older patients did not show any benefits in terms of survival, improvement in quality of life, or reduction in aspiration pneumonia. Significant gaps exist regarding the evidence for the evaluation and management of dysphagia in older patients. This study aimed to diagnose swallowing disorders and highlight the importance of swallowing therapy in older patients in intensive care units (ICUs).

**Materials and methods:**

Twenty-five older patients (12 men, 13 women, mean age 67.22 ± 24.03 years) hospitalized in the ICUs with complaints of dysphagia were analyzed prospectively. The 12 weeks (14−16 sessions) of swallowing therapy were administered to patients with dysphagia who signed the (voluntary) consent form. The bedside water swallowing test (BWSS), Functional Oral Intake Scale (FOIS) Score, Clinical Swallowing Evaluation, Mini Nutritional Assessment Test (MNAT), Eating Assessment Tool (EAT-10), the Turkish version of the World Health Organisation Quality of Life Scale Elderly Module, and the Swallowing Therapy Programme Protocol were applied. Pretherapy stage (T1) and post-therapy stage (T2) results were compared with videofluoroscopy swallowing study (VFSS) recordings with thin liquids, moderately thick liquids, extremely thick liquids, and crackers (International Dysphagia Diet Standardization Initiative [IDDSI] Levels 0, 3, 4, and 7, respectively).

**Results:**

The World Health Organisation Quality of Life Scale Elderly Module (WHOQOL-OLD) raw scores significantly improved from T1 (38.63 ± 7.05) to T2 (73.07 ± 4.82). The bedside water swallowing test results demonstrated statistically significant differences in therapy timings among older patients (*p* < 0.001). There were significant improvements in swallowing physiology, as represented by the improved oral and pharyngeal composite scores of the Modified Barium Swallow Impairment Profile (MBSImP) and Penetration-Aspiration Scala (PAS) levels. Before therapy, all patients exhibited high rates of oropharyngeal residue with thin liquids and spoon-thick pudding viscosities (MNA ≤ 17). The results reported in the present study show that malnutrition risk is linked to a poorer QoL in older patients on admission to ICUs. Statistical analyses revealed the dominant effects of functional status and eating-related factors on QoL in this group.

**Discussion:**

Early dysphagia diagnosis of older patients and subsequent application of exercise-based swallowing therapy increase the quality of life of patients. In this study, exercise-based swallowing therapy was developed in Turkey and can be used in older patients as part of a holistic cognitive-communication-swallowing intervention program. Results prove the effectiveness of the developed exercise-based swallowing therapy on the cognitive-communication-swallowing skills of older patients. The present findings reinforce the role of nutrition as a priority for improving patients’ perceptions of QoL. Further studies are required to investigate and identify the interventions that improve QoL in older patients. More studies with better research designs are required to establish whether nutritional intervention is effective in enhancing QoL in this vulnerable group.

## Introduction

Estimates of dysphagia prevalence vary widely among older patients due to differences in assessment methods and healthcare settings; the average prevalence is approximately 15% among community-dwelling older patients (1) and approximately 47.4% among patients admitted to acute geriatric care (2). In older patients with dysphagia, particularly oropharyngeal dysphagia [OD], it is a common and detrimental complication (3).

Dysphagia is swallowing difficulty that may occur due to oropharyngeal or esophageal problems in older patients (2, 3). Swallowing disorders hinder food consumption, often resulting in weight loss, malnutrition, and dehydration. The commonly reported symptoms in these patients would be the pocketing of food in the mouth, difficulties with mastication, coughing or choking with food or fluid, and the need for reminders to swallow food. Some of the contributing factors to oral phase dysphagia include the inability to recognize food visually, oral-tactile agnosia, and swallowing and feeding apraxia (2, 3). In older patients, pharyngeal phase dysphagia leads to aspiration before, during, and after swallowing. Aspiration pneumonia has further been reported to be a cause of death in older patients (3, 4). We recently found that the prevalence of OD in independently living older subjects (>70 years) was 27.2% (5), and other studies have found a prevalence of over 51% in older institutionalized patients, many of them tube-fed due to severe swallowing dysfunction (6).

Despite the growing number of older patients who suffer from dysphagia, only a few studies are reporting the evaluation and management of older patients (2, 3). This study aimed to evaluate swallowing function in older patients hospitalized in ICUs and to diagnose swallowing disorders.

Hospital malnutrition, typically defined as a deficiency in energy or one or more nutrients due to illness, injury, or inadequate intake, is commonly observed in developed countries, contributing to negative health outcomes and increased healthcare costs. Timely identification and management of malnutrition are crucial. The lack of a universally accepted definition and standardized diagnostic criteria for malnutrition has led to the development of various screening tools, each with varying validity. This complicates the early identification of malnutrition, hindering effective intervention strategies (7). Differences in the definitions and diagnostic criteria of malnutrition have led major clinical nutrition societies to define their own parameters. The American Society for Parenteral and Enteral Nutrition (ASPEN) (8) diagnoses malnutrition with two or more parameters such as insufficient energy intake, weight loss, loss of muscle mass or subcutaneous fat, localized or generalized fluid accumulation, and diminished functional status as measured by handgrip strength. In contrast, the European Society of Parenteral and Enteral Nutrition (ESPEN) requires any three of five parameters, including body mass index (BMI), weight loss, recent food intake, muscle mass, and disease severity (9).

Other objectives of the study were to determine the presence of residues and penetration-aspiration frequency and reveal the relationship between dysphagia and quality of life by creating a personalized swallowing treatment plan through early diagnosis, which aims to improve patients’ quality of life.

This study hypothesizes that in older patients with dysphagia, the functional swallowing results and quality of life results after swallowing therapy will be ‘better.’ In this study, to make an early diagnosis, the swallowing function of individuals was determined through clinical swallowing and instrumental evaluation and bedside water swallowing test (BWST), nutritional status was evaluated, and the effect of the results on quality of life was investigated. In short, the goals of this study were as follows:

- To develop a swallowing therapy program that includes a holistic cognitive–communication–swallowing–nutrition intervention program for both cognitive and communication disorders that can be used in older patients.
- To examine the effect of the developed therapy program on patients’ swallowing and communication skills.
- To compare the nutrition changes with therapy times among individuals with older patients in the study group.

What this paper adds:

It has been established that there are a few (as far as is known, two) holistic therapy swallowing programs developed in English for cognitive-communication-swallowing disorders due to older patients in ICUs.

What this study adds to the existing literature:

In this study, for the first time in Turkish, a holistic therapy swallowing program has been developed for cognitive-communication, especially swallowing disorders, that is specific to older patients in ICUs and can be used by individuals at advanced stages of the disease. The effects of the holistic therapy swallowing program we developed on the swallowing skills of individuals with older patients were shown in a randomized trial. In this context, our study is the first attempt to show that an exercise program is feasible in older patients in ICUs.

What are the potential or actual clinical implications of this work?

Using the holistic therapy swallowing program can help people with older patients maintain their cognitive, communication, swallowing, and nutritional skills and have a better prognosis in terms of cognitive-communication-swallowing skills, especially oral intake, and quality of life.

Main Points:

- Dysphagia is common in older patients and is one of the common clinical geriatric syndromes.
- Bedside and clinical swallowing and instrumental evaluation grades, in addition to the swallowing therapy grade of the older patients who suffered from dysphagia and who stayed in the intensive care unit.
- Swallowing functions and nutritional status (NG, PEG, oral intake, and more) deteriorate as the disease progresses. Individuals should be evaluated in terms of malnutrition and dysphagia; early preventive and therapeutic strategies should be developed to prevent conditions such as aspiration pneumonia that may develop in the future, and the patient and his family should be guided.
- Older patients hospitalized in the intensive care unit, cognitive and behavioral disorders are mostly emphasized, and the problem of dysphagia is generally ignored.
- Older patients in ICUs should be inspected in detail in the initial stage in terms of dysphagia, and exercise-based swallowing therapy-dysphagia treatment should be applied with necessary preventive measures.
- To establish whether nutritional intervention (therapeutic nutrition therapy in a holistic swallowing therapy program) is effective in enhancing QoL in this vulnerable group—older patients—more studies with better research designs are required.

## Methods

### Trial design

This prospective study was carried out in the Ankara Bilkent City Hospital Intensive Care Unit (ICU) with the approval of the Ankara Bilkent City Hospital Clinical Research Ethics Committee (Decision No: E1-23-3185). All procedures performed in this study involving human participants were in accordance with the ethical standards of the institutional and/or national research committee and with the 1964 Helsinki Declaration and its later amendments or comparable ethical standards.

#### Informed consent with older patients

Getting informed consent from older patients depends on their mental capacity. Geriatric symptoms, like difficulties with concentration and understanding problems in short-term memory, make their ability to give informed consent questionable. These symptoms become more of a problem as the disease progresses. It would be appropriate to involve relatives if an older patient cannot consent. However, it is necessary to understand that being an older patient does not mean that a person cannot consent. Therefore, conducting a mental capacity assessment for older patients is important. When evaluating a patient who may lack mental capacity, one must apply the following five statutory principles of the Mental Capacity Act (MCA) (10).

It is advised that consent be gained each time we interact with older patients for research or treatment purposes rather than just once. This is to overcome short-term memory problems and variable capacity. It is important to always make sure to obtain consent from older patients. Two main values govern the need to obtain informed consent: (1) to promote and protect the person’s wellbeing and (2) to respect the person’s self-determination (10).

It has been noted that even an older patient with clearly impaired capacity can still indicate a choice and show some understanding. The four key decision-making components in a capacity assessment are understanding, communicating a choice, appreciation, and judgment. Assessment of capacity requires a direct interview with the patient using open-ended questions and can include both informal and formal approaches depending on the situation and context (10).

The purpose of the study was explained to the participants and caregivers, and written informed consent forms were obtained using appropriate forms. We have consented each time we interact with older patients hospitalized in the ICU for research or treatment purposes, rather than just once in this study. Capacity assessment requires a direct interview with older patients hospitalized in the ICU using open-ended questions. It also includes both informal and formal approaches depending on the situation and the context of this study. In terms of adherence and dosing considerations, before the patients were instructed to complete the exercises, the clinician demonstrated the exercise as an example (3 sets) (clinician dependent), then completed it with the help of the caregiver (2 sets) (clinician dependent). Finally, the case was encouraged to do it herself after five repetitions by both the SLP and the caregiver and nurse. After these processes, she was able to do the exercises without total assistance. In cases where information from the patient’s relatives was required, information was obtained from a family relative or caregiver who cares for the patient for at least 20 h a week and can provide reliable information about the patient’s functional status.

#### Evaluation-primary outcomes, secondary outcomes

Bedside and clinical swallowing and instrumental evaluation grades were evaluated, as well as the swallowing therapy grade of older patients who suffered from dysphagia and were hospitalized in the intensive care unit. The bedside water swallow test was performed the day before the videofluoroscopic swallow study (VFSS) and was used as a screening measure to determine eligibility for the BWST. We defined older patients with “cognitive dysfunction,” but that can follow the Holistic Swallowing Therapy Programme Protocol (sensory stimuli, strategies, exercises, manoeuvres, and so on). We studied a treatment effect prospectively. Adverse events and unexpected symptoms during the intervention will be recorded and analyzed. The safety coefficient of each group is calculated as follows. Safety coefficient = (the number of adverse events/the number of engaged participants) *100%. Before commencement, the nurses and research assistants will be instructed about the safety considerations and safety protocol to avoid the risk of medical emergencies during the implementation. The caregivers or family members will respond to the questionnaire, while the participants undergo the cognitive function test. Participation in the study will provide no direct benefit to the respondents.

#### Study setting and participants

These individuals’ files were reviewed, and participants who met the inclusion and exclusion criteria were identified.

The inclusion criteria in the study were the older patients and the presence of complaints of dysphagia. Hospitalization information, current illnesses, and patient diagnoses were searched and grouped (3). Older patients with dysphagia were included in the study group through clinical swallowing evaluation.

**Figure 1:**
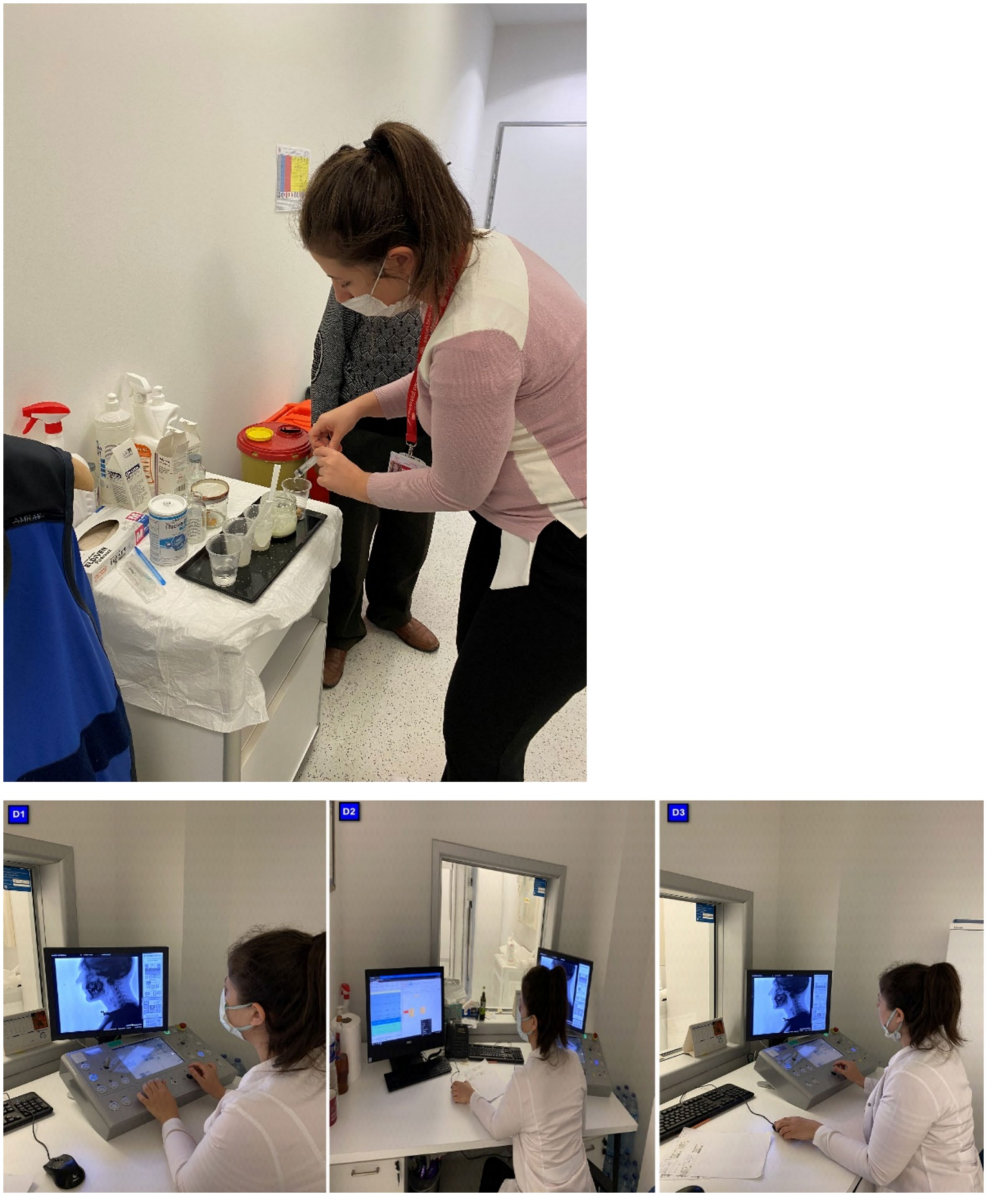
Flow chart of the trial.

This study evaluated swallowing efficiency and safety by evaluating 10 mL of bedside water swallowing. In the BWST used in the clinical evaluation (Figure 1), saturation was monitored using pulse oximetry (11). The SLP was called to the patient in the radiology unit for simultaneous VFSS while the patient underwent a routine Magnetic Resonance Imaging (MRI). Blinded raters scored the VFSS recordings for safety and Modified Barium Swallowing Impairment Profile (MBSImP) metrics in an efficiency-randomized fashion to examine swallowing function and physiology changes from baseline to the posttherapy stage.

#### Sample size

The sample size was determined using the comparison of BWST. The required sample size for the study was determined to be the G*Power program 3.1 software. When alpha was 0.05, the power value was 0.80, and the effect size was 0.52 (Cohen’s f), the sample size was determined to be 25.

Inclusion criteria are (a) diagnosed as older patients by The World Health Organization, which has specifically classified both conditions using the International Statistical Classification of Diseases and Related Health Problems ICD-9 and ICD-10 (Oropharyngeal Dysphagia: 787.2, R13, and Malnutrition: 260–69, E40-E46); (b) score a third degree or higher on the BWST, demonstrating a risk of aspiration; (c) the MNA recommended by the ESPEN for the screening of MNAT in older patients (12) and (d) have basic communication ability to complete the tests in this trial. We obtained information on the route (enteral and parenteral) and amount of nutritional support (protein and calories) provided daily from the electronic medical records by the lead dietitian in the malnutrition unit in the intensive care team. As indirect calorimetry was not available, energy and protein targets (25 kcal/kg/day and 1.3 g/kg/day, respectively) were calculated according to real or adjusted body weight in agreement with the recent European guidelines (13). Early caloric and protein deficit was defined as the incapacity to reach 80% of the estimated caloric and protein requirement in the first month (14).

Protein undernutrition enhances frailty and aggravates intercurrent diseases generally observed in older patients. Undernutrition results from insufficient food intake and catabolic status. Nutrient intake was lower in older patients hospitalized in short-stay care units, perhaps due to failure to recognize suitable nutrient requirements. Protein-caloric undernutrition should be diagnosed early during hospitalization to allow appropriate dietary supplementation.

Daily nutrient intakes were explored for hospitalized older patients. In this study group, before therapy, older patients in ICU had lower total caloric intake, lower protein intake, and dramatically lower calcium intake (total caloric intake: 1365 +/− 500 Cal/day; protein: 0.8 +/− 0.3 g/kg/day; carbohydrates: 53 +/− 8.7%; lipids: 32 +/− 6.2%; calcium: 854 +/− 263 mg/day).

Malnutrition was determined in all the patients according to the Gomez classification. The Gomez classification system was used to determine the degree of malnutrition, and patients were divided into three groups: mild, moderate, and severe. The patients were further classified by height for age and weight for height according to the Waterlow classification to evaluate the patients in terms of acute and chronic malnutrition.

Anthropometric measurements were used to evaluate the nutritional status of the patients.

Anthropometric measurements included the body mass index (BMI ¼ weight in kg/height in m2), the percent weight loss, and the time over which the weight loss was collected.

Biochemical nutritional markers included total protein (reference values: 6.0–8.3 g/dL) and cholesterol (120–200 mg/dL).

Mid-upper arm circumference (MUAC) was measured using a flexible nonstretch tape to the nearest 0.1 cm at the marked midpoint (between the acromion and olecranon process) on the non-dominant arm (15).

Within 4 months, 85 individuals with older patients were hospitalized in the ICU. The files of these individuals were examined, and 31 individuals who met the inclusion and exclusion criteria were found. According to the post-test results of these individuals, it was determined that 25 had a dysphagia risk. Among those who met the inclusion and exclusion criteria, study group participants were randomly selected. Randomization used the stratification technique. A total of 25 older patients were randomly selected for the study groups, for a total of 25 participants. Patients and relatives were informed about the study and invited to the clinic for evaluation. Since there were patients with regular follow-up, no one refused to participate in the study among the 25 patients.

Swallowing evaluations were performed on 25 intensive care patients with older patients (12 men, 13 women, mean age: 67.22 ± 24.03 years) suffering from dysphagia. Therapy sessions were held once, three sessions per week (25–45 min), during the 12-week holistic exercise program for each patient with dysphagia who signed a voluntary consent form in the bedside assessment and therapy in the intensive care unit. A guardian’s relatives /the participants’ caregivers also signed the consent form. The participants, along with older patients, consented to the study. They had the capacity to give informed consent. The voluntary nature of the study was explained in the cover letter, which was sent out to all experts and endusers. We considered receiving a response as consent was given. A total of four cases could not tolerate VFSS. Two cases did not complete follow-up. A total of 25 participants were able to tolerate the videofluoroscopy procedure and follow the instructions. A full clinical swallowing evaluation was appropriate for all participants, and the assessments were tolerated. Interventions were possible in a prospective setting.

**Figure 2:**
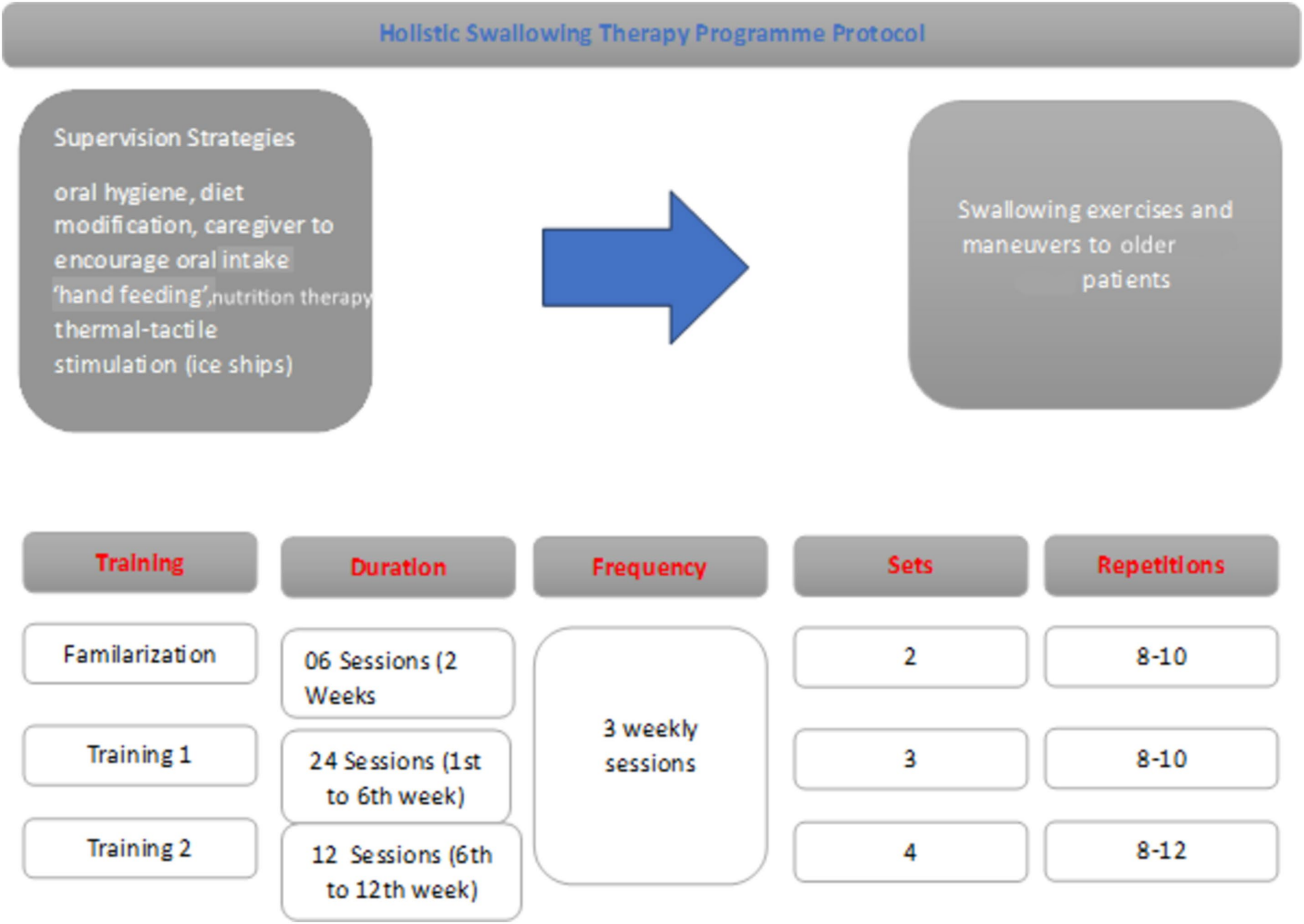
WHOOQL-OLD total comparison based on therapy timings. In our study, when comparing the intertemporal WHOQL-OLD raw scores, it was 73.07 ± 4.82 at T2 time and 38.63 ± 7.05 at T1 time (Figure 2).

Figure 2 depicts the distribution of the individuals in the study.

The results of dysphagia in older patients were compared before therapy (T1 time) and after posttherapy (T2 time) by a speech and language therapist (SLP).

Severe psychiatric conditions (schizophrenia), intellectual disability, primary progressive aphasia, being diagnosed with one of the other types of dementia, having previously participated in any cognitive and/or communicative therapy, having a history of additional neurologic disease (stroke, Parkinson’s disease, etc.), having a history of sarcopenia and sarcopenic dysphagia, being allergic to barium, not eating/drinking some liquids and/or solids by mouth, and being unable to attend follow-up sessions were the criteria for exclusion and withdrawal from the study. Misunderstanding of common ICU problems: Intubation is known to be a primary risk factor for dysphagia in ICU patients, distinguishing it from oropharyngeal dysphagia. Delirium is a prevalent geriatric syndrome among ICU patients, particularly those with cognitive impairments (16). It is crucial to assess patients for delirium instead of cognitive tests in ICU settings. Therefore, the delirium evaluation factor is our exclusion criterion to ensure data validity in our study.

### Recruitment

A timeline was prepared showing the procedures’ completion order and the time elapsed between procedures.

**Table:** 

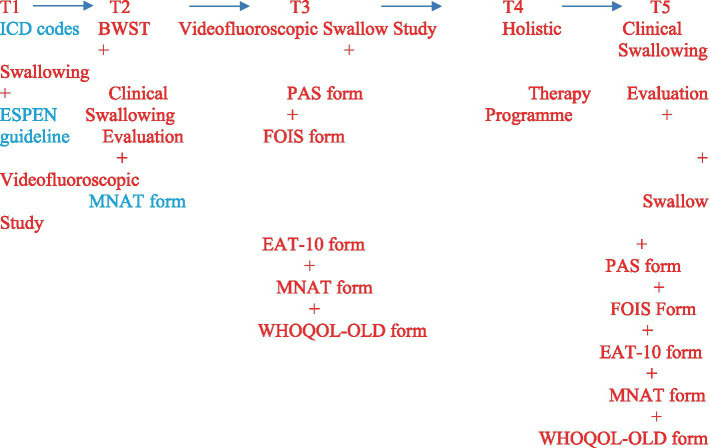

#### The bedside water swallowing test (BWST)

BWST is frequently used in clinical practice as a functional assessment to detect aspiration and prevent pneumonia. It is a standardized test used worldwide, but the amount of water given varies depending on the examiner. In our study, six conditions were evaluated, including the presence of cough and bifurcation of the voice during and within 1 min (min.) after drinking 10 millilitres (ml) of water, the presence of aspiration, splitting during drinking, water discharge from the corner of the mouth, absence of laryngeal movement, and a decrease in oxygen saturation of 2% or more during and within 10 min. After drinking the water. The presence of each of these six conditions was assigned a score of 1 and 0, and the total score was interpreted as normal swallowing if the score was between 0 and 2 and dysphagia if the score was between 3 and 6. This test will be used to determine the effectiveness of the Holistic Program. Evaluators observed individual reactions after drinking 30 mL of water (complete recovery: swallowing without cough; improved swallowing function: occasional choking, and invalid: unchanged or worse swallowing function). Effective rate = (completed recovery + improved swallowing function)/total cases in each group *100%. The sensitivity and specificity of this test ranged from 34.8 to 55.7% and 78.9 to 93.2% in older patients (17).

This test is a useful adjunct to a clinical examination, helping to highlight patients who require an instrumental assessment such as videofluoroscopy. Furthermore, quantitative measures can be derived from this test, which can be used as a measure of swallow performance over time.

#### Videofluoroscopic swallowing evaluation

**Figure 3:**
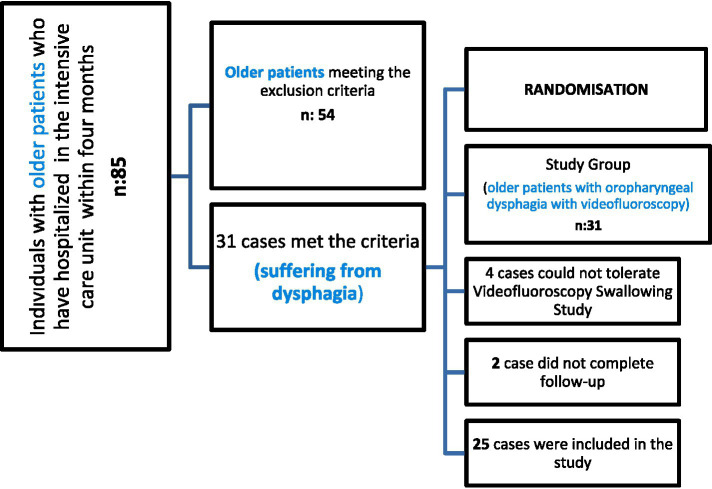
HSTP flow chart. We have given detailed information about training times, duration, frequency, sets, and repetitions in Figure 3.

Videofluoroscopic swallowing was evaluated in the Ankara Bilkent City Hospital, Department of Radiology, General Hospital, General Radiology, and Fluoroscopy room. CONNEXITY GE, 01144/TAEK77555 Fluoroscopy System (GE Healthcare Medical Systems, France 2012) was used, and VFSS recordings were taken at 25 frames per second (fps). The Velopharyngeal Cinefluoroscopy procedure with the 802.330 ICD code must be made by the Medical Oncology Unit before the protocol (Figure 3). In the protocol, lateral and anteroposterior imaging were performed on the individuals. Before starting the videofluoroscopic swallowing evaluation, the materials to be used during the test were prepared according to the criteria determined by IDDSI. Then, the procedure to be applied to the individuals was explained in detail, and information was given about what was expected of them. Management of the Dysphagia Plan was performed. Before barium contrast (impregnated food and liquid as they are transported during the oral cavity, pharyngeal cavity, and oesophagus in real time) was given to the patient, the Glomerular Filtration Rate (GFR) value was checked, which is a procedure of our hospital. In accordance with accepted definitions, oral and pharyngeal VFSS signs of safety (penetrations, aspirations, Rosenbek’s penetration-aspiration scale) and efficacy of deglutition (mainly residue) were identified.

##### Preparation of test materials

###### Bolus trial (IDDSI)

In our study, the consistency types and bolus volumes specified by Dr. Steele in his pioneering studies were used (18, 19).

This study aimed to profile the number of swallows elicited in older patients across thin, moderately thick, and extremely thick, and crackers (International Dysphagia Diet Standardization Initiative [IDDSI] Levels 0, 3, 4, and 7, respectively). Research speech-language pathologists performed VFSS using a standardized bolus protocol and 40% w/v concentration of Varibar barium sulfate products (Bracco Imaging), which have been mapped to IDDSI levels (18).

Swallows from the standardized bolus protocol that were used in the analyses for this study included three cued 5-cc thin liquid boluses via a 30-cc medicine cup (IDDSI Level 0), one uncued comfortable cup sip of thin liquid from a cup filled to 90 cc (IDDSI Level 0), three cued 5-cc thin honey boluses by spoon (IDDSI Level 3), two cued 5-cc pudding trials (IDDSI level ≥ 4) by spoon, and one-fourth graham cracker coated with pudding (IDDSI Level 7) (18).

To maintain patient safety during VFSS examinations in our study, VFSS was discontinued if research participants exhibited three episodes of gross aspiration (> 25% bolus volume swallowed) and/or if they exhibited >75% pharyngeal residue in the pharynx despite efforts to clear it (20).

##### Interpreting the shots

The recordings were recorded in the fluoroscopy system, taken from the system with an external hard disc, and viewed later using the Radiart software. A PAS score of 1 indicates no penetration or aspiration during swallowing and that the normal airway is preserved. Scores of 2–5 are considered penetration, indicating that the bolus has entered the larynx but not below the vocal folds. Scores between 6 and 8 are considered aspiration, meaning food has passed under the vocal folds. Penetration-aspiration scale level was determined for each consistency at the same time as the PAS scoring was done, as recommended by the protocol. Total scores were calculated and recorded. To assess the reliability of test interpretation, 20% of all tests were interpreted by a second language and speech therapist who is blind to all other variables. In terms of the reliability of the analyses, a correlation was determined between the findings of both evaluators.

#### PAS (penetration and aspiration scale) form

It is important to determine the penetration and aspiration severity in the evaluation of the treatment indication in swallowing disorders. To increase the power of instrumental techniques, various rating scales have been developed to express clinical status and aspiration severity. PAS provides information about the presence and severity of aspiration and penetration. Our study prefers it because it is widely used in the clinic. PAS, which determines the severity in patients with aspiration and penetration, rates the penetration aspiration score between 1 and 8 (11). According to this scale, 1 point means no penetration and aspiration, 2–5 points mean penetration, and 6–8 points mean aspiration (11).

Age, sex, and hospitalization diagnosis information were obtained for all cases, and the presence of dysphagia was recorded. We diagnosed dysphagia if the patient had penetration and/or aspiration with or without symptoms. Also, delayed swallowing reflexes, oropharyngeal phase problems, motility problems, and nasal regurgitations were recorded according to VFSS.

The researchers carefully considered Steele and Grace-Martin’s article on statistical methods for the Penetration-Aspiration Scale at the beginning of this study and used it as a guide (21).

#### FOIS (functional oral intake scale) form

The Functional Oral Intake Scale (FOIS) shows the functional levels of the patients in their diet (11). It grades the functional level of oral intake in 7 steps according to the solid and liquid food that individuals take orally. The level of the functional oral intake scale is determined by questioning the oral intake status of the patient (1–2-3: non-oral nutrition, 4–7: oral nutrition, 7: normal) (11). The FOIS score grades the functional level of oral intake in seven steps based on the solid, semi-solid, and liquid foods that individuals consume orally. The levels of FOIS were determined by questioning the oral intake status of the patient (1–3: non-oral nutrition; 4–7: oral nutrition; >7: normal) (11).

#### Clinical swallowing evaluation by SLP

The main complaints of the patients were the onset of their complaints and their progression, as well as the food types of their complaints. How they appeared, possible pneumonia history, eating habits, and reflux symptoms were recorded. Nutritional style, diet level, presence of a tracheostomy tube, presence or history of an endotracheal tube, need for aspiration (suction), complaints such as coughing during eating and drinking, voice change, feeling of suffocation and choking, weight loss, malnutrition, adequate fluid consumption, physical condition, and additional barriers were examined. The patient’s respiratory status, blood oxygen saturation level, respiratory rate, and need for respiratory support were recorded.

During physical examination, the condition of the anatomical structures in the oral cavity, oral hygiene, moisture in the mouth, and lesions were observed during oral cavity observation. The intraoral sense was evaluated, and the amount of mouth opening was measured. The strength of the tongue, lips, and facial muscles was evaluated by oral motor evaluation, and the oropharynx was examined by looking at the presence of the gag reflex, movements of the velum and uvula at rest, and the phonation state by SLP (12).

After the laryngeal examination was performed in the clinical setting, the duration of the patient’s ability to maintain phonation, quality of the voice in phonation, intensity of the voice, presence of the swallowing reflex by palpation during dry swallowing, and the amount of laryngeal elevation were examined. A cranial nerve (CN) examination was performed during the oral motor examination. Jaw movements and the state of the masticatory muscles, the state and strength of the facial muscles at rest and in motion, lip movements, and lip closure strength were evaluated, and the presence of gag reflex, velar movement, phonation, and voluntary cough were examined. The SLP also examined the resting state of the tongue (unilateral or bilateral atrophy, fasciculation, and spasticity), tongue range of motion, and tongue muscle strength (12). All information from patient files was recorded from the SLP’s therapy form. The following criteria were considered in the evaluation in terms of nutrition education and pulmonary aspiration (during enteral nutrition for 48 h postoperatively): oral care with antiseptics at least twice a day, keeping the head height 35−40° above the bed floor, increasing the height of the bed head 30−60 min when feeding is interrupted, follow-up of the patient in terms of symptoms such as nausea-abdominal pain-distension, and confirmation of tube location with the radiological image (from patient file records) (22).

In each procedure, the patients’ oxygen saturation levels (sPO2) and pulse status (over and under 40 times per min) were continuously controlled. The patient’s oxygen saturation level (SPO_2_) and pulse status (over and under 40 times per min) were continuously controlled in each procedure. A BWST was performed (23). Six patients were evaluated for the presence of coughing and wet voice after aspiration while drinking 10 mL of water and, within the next 1 min, splitting the water while drinking, water discharge from the mouth, absence of laryngeal movement, and reduction of ≥2% in oxygen saturation while drinking water and within the next 10 min.

For each item, the authors obtained explanatory information from the relevant nurse and caregiver beforehand to ensure that the questions in the EAT-10 were understood and that the cognitive level did not affect the patient’s reports of symptom severity.

#### Eating assessment tool (EAT-10) form

The EAT-10 is a self-scoring scale that scores the patient’s perceived extent of dysphagia symptoms. The total score obtained from the questionnaire is an important guide for the patient’s swallowing disorder. While applying the EAT 10, which can be applied in a simple, easy way that allows us to obtain information about predicting the risk of aspiration, a total of 10 questions were asked. For each question, responses were scored from 0 to 4, ranging from no problem to severe problem. If the results were 3 and above 3, it was considered dysphagia; if below 3, it was considered normal swallowing (23).

##### Holistic swallowing therapy program protocol study design

This randomized trial was carried out in four stages:

Phase 1: Baseline data collection: Scale rating.

Phase 2: Participants’ evaluation and practical training.

Phase 3: Participants use the Holistic Swallowing Therapy Programme Protocol at ICUs.

(Follow-up period):

-T1: Intervention completed-PRETHERAPY STAGE.
-T2: Twelve weeks after intervention-POSTTHERAPY STAGE.

Phase 4: Participants’ re-evaluation: Data analysis.

#### Holistic swallowing therapy program protocol

**Figure 4:**
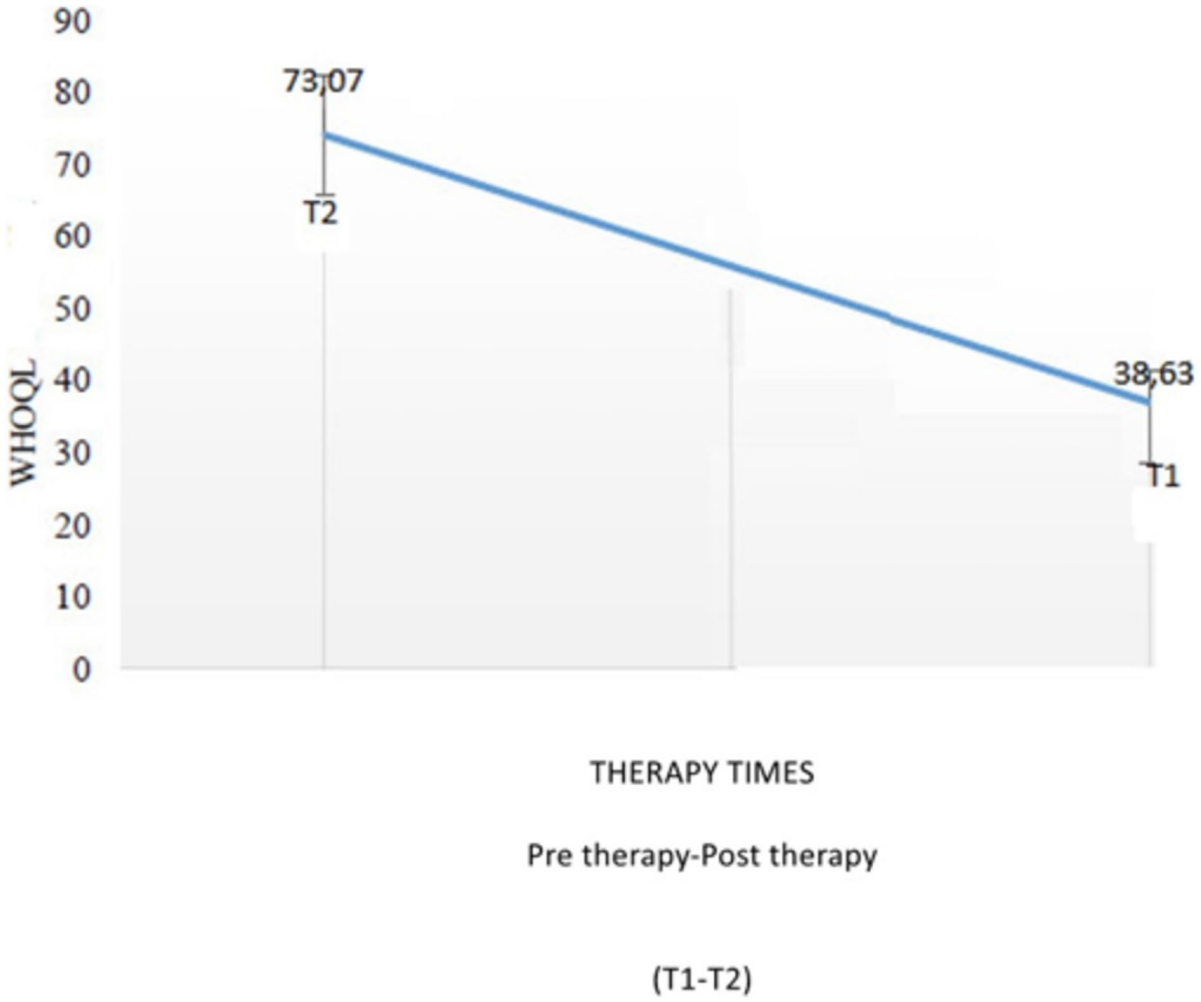
Clinical swallowing evaluation of IDDSI level 4* in the intensive care unit. *Three cued 5-cc thin liquid boluses via a 30-cc medicine cup (IDDSI Level 0), one uncued comfortable cup sip of thin liquid from a cup filled to 90 cc (IDDSI Level 0), three cued 5-cc thin honey boluses by spoon (IDDSI Level 3), two cued 5-cc pudding trials (IDDSI level ≥ 4) by spoon, and one-fourth graham cracker coated with pudding (IDDSI Level 7). *Steele et al. (18).

Sensory stimuli, strategies, exercises, and manoeuvres used in the exercise-based intensified swallowing therapy program in our study are given below: these are oral hygiene, diet modification, caregiver encouragement of oral intake ‘hand feeding’, thermal-tactile stimulation (ice chips), effortful swallowing, the Mendelsohn manoeuvre, the Masako manoeuvre, supraglottic swallowing manoeuvre, the super supraglottic swallowing manoeuvre, modified shaker exercises and effortful pitch glides (24, 25). Therapy sessions were held once, three sessions per week (25−45 min) during the 12-week holistic exercise program for each patient in the bedside assessment and therapy in the intensive care unit (24, 25). We have given detailed information about training times, duration, frequency, sets, and repetitions in Figure 4. Moreover, when participants are discharged from the hospital, training videos and anime pamphlets are given to the nurses, family members, and caregivers.

##### Routine nursing care during intervention in the ICU

During the intervention and follow-up, participants in the study group and their caregivers received routine nursing care and health education about swallowing dysfunction.

##### Therapeutic nutrient therapy

Therapeutic nutrition is a speciality that uses diet to treat or manage various diseases and health conditions. During the intervention and follow-up, participants in the study group were received by a registered ICU dietitian. The nutrition therapy used in our study included behavioral and lifestyle changes, such as changing one’s eating habits, eating a balanced diet, and leading an active lifestyle. The registered dietitian built a plan tailored to our older patients’ needs, conducted nutrition assessments, and identified and documented malnutrition based on AND/ASPEN guidelines (26–28).

##### Positioning

- *Thermal-Tactile Stimulation:* Sour tastes are known to be effective in triggering swallowing. It has been stated that sufficient studies suggest the effect of odor perception and food texture perception on triggering swallowing. Mechanical stimulation, cold, and taste perception stimulation have been shown to have less effect on swallowing delay in healthy individuals. The study triggered the swallowing reflex by stimulating the anterior faucial arches (anterior neck region) with cold. We thought that this facilitated the oro-pharyngeal phase.
- To decide which compensation to use, videofluoroscopic imaging techniques were used to ensure that the airway was completely closed.

###### Supraglottic swallowing manoeuvre

The patient was asked to sit at a right angle, take a deep breath, and hold his/her breath. While holding his breath, he was asked to swallow and cough after each swallow. It is preferred for patients with decreased vocal cord closure.

###### Effortful swallowing manoeuvre

Sitting upright on a chair, the patient was asked to compress all the mouth muscles during swallowing. The caregiver and intensive care nurse were always with us while the command was being explained. With this manoeuvre, tongue root retraction was increased, and delayed swallow triggering occurred.

###### Mendelsohn manoeuvre

In this manoeuvre, the patient must realize that he/she is doing a laryngeal elevation (raising the larynx). Supported by visual materials, the SLP and the nurse do it with the patient and the caregiver as role models. The patient was asked to bring the larynx to the highest possible level and hold it for a few seconds. The patient is then asked to relax, and swallowing is completed.

###### Tongue lift manoeuvre (masoko manoeuvre)

The patient is asked to hold the tongue between your front teeth and swallow. Modified Shaker Exercise.

To overcome the disadvantages of the shaker exercise, we modified the position to supine, lying at a 45° inclination. Forty-five-degree inclined lying position.

The head and neck posture was positively affected by increasing body awareness and neck range of motion in older patients who could not use their necks functionally. For this reason, the modified position was used as an alternative in these geriatric individuals and in people who had difficulty with the Shaker exercise (What is done in the Shaker exercise is repeated with 45-degree back support).

##### Effortful pitch glides

1. To ensure airway closure, the patient sitting in the chair holds his/her breath and pushes the chair down with his/her hands with elbows open and straight.
2. The person sitting in the chair was asked to push the chair down with both hands with elbows straight out and say ‘ahhhh’ or ‘iiiiii’ for at least 5 s.
3. Asked to yawn or gargle by pulling the tongue back as straight as possible. (This should be done without food in the mouth). The caregiver’s support is requested when positioning the patient.

#### The method and content of nutrition during the ICU stay

The European Society for Swallowing Disorders (ESSD) position statements declare that OD is a risk factor for malnutrition. A nutritional examination should be performed regularly using validated nutritional screening tools (29). In addition, the ESPEN guidelines on nutrition in older people recognized OD as a major cause of impaired nutritional intake among this population. Despite all these recommendations, both conditions continue to be underestimated and underdiagnosed and can be considered neglected conditions among older people (29, 30).

##### Clinical assessment of Malnutrition

###### Mini nutritional assessment test (mini nutritional assessment) (MNA)

The form of the Mini Nutritional Assessment Test (Mini Nutritional Assessment) (MNA) was applied to screen malnutrition. MNA is comprised of 6 items, including weight loss, body mass index (BMI), acute diseases, mobility, mental disease, and dysphagia or anorexia. Patients who scored less than 17 in the MNA were considered malnourished, and those between 17 and 23.5 were considered at risk of malnutrition. The specificity and sensitivity of MNA in predicting malnutrition were 88.8 and 85.6%, respectively (31).

###### World Health Organization quality of life scale elderly module Turkish version (WHOQOL-OLD)

World Health Organization Quality of Life Scale Elderly Module Turkish Version (WHOQOL-OLD) was administered. In the WHOQOL-OLD module, the lowest possible score for each question is 1.0, and the highest 24 questions were asked with a high score of 5.0. Sensory ability, social participation, death and dying, bonds of intimacy, autonomy, and the past, present, and future are six sub-areas of activities that were assessed. For each subsection, the patient, or according to information from his family, quality of life is directly proportional to the increase in 4–20 points between the six domains. When calculating the raw score, the total score of 6 fields was calculated.

### Statistics

The Shapiro–Wilk test was used to evaluate whether the variables in the study were in accordance with the normal distribution. Median (Minimum-Maximum) values are given in the display of descriptive statistics of the variables. Number (n) and percentage values were also given in the representation of variables such as therapy time and number obtained within the scope of the study. In the representation of descriptive statistics of normally distributed variables, mean (mean) and standard deviation (SD), median (median), and interquartile range (CIW) were used to display descriptive statistics for non-normally distributed variables. A paired *t*-test and Wilcoxon ordinal number of signs test were used to determine whether the total variables for EAT-10, water drinking, and WHOQOL-OLD showed statistically significant differences between T1 and T2 times. IBM SPSS Statistics 26.0 program was used, and the statistical significance level was accepted as a *p*-value of<0.05.

Spearman’s rank correlation coefficient was used to test the association between WHOQOL-OLD indices and nutritional markers. WHOQOL-OLD scores for those scoring above and below thresholds for nutritional risk were compared. Regression models were created to identify nutritional indices contributing to the variability of WHOQOL-OLD.

## Results

The mean age of the study participants was 67.22 ± 24.03, with a minimum age of 67 and a maximum age of 81. A total of 25 older patients [12 men (48%) and 13 women (52%)] participated in the study (Table 1). All participants completed the Swallowing Therapy Program (100%).

**Table 1:** Demographic information of the cases, stage type, average length of stay in the intensive care unit, additional comorbid diagnoses and swallowing therapy programs.

Parameters	Number (Percent) *n* (%)
Gender	
Women	13 (52%)
Men	12 (48%)
Age (mean ± SD; years)	67.22 ± 24.03
Educational status	
No reading or writing	17(68%)
There is reading and writing	8(32%)
Patients	
Older patients with oropharengeal dysphagia	25(100%)
Hospitalization time in an intensive care unit	27.22 ± 25.03

The mean age of the individuals participating in the study was 67.22 ± 24.03, with a minimum age of 67 and a maximum age of 81. A total of 25 older patients with OD [12 men (48%) and 13 women (52%)] participated in the study (Table 1).

MNAT results showed a significant statistical increase after the therapy in our study. In the WHOQOL-OLD quality of life evaluation of our study, the values of 4 parameters, including sensory, autonomy, past, present, and future activities, and social participation, increased gradually toward the T2 (post-therapy) stage, and this increase was statistically significant between the stages (Table 2).

**Table 2:** Comparison of Mini Nutrition Assessment Test (MNAT) and World Health Organization Quality of Life Scale Elderly Module Turkish Version (WHOQL-OLD) values of older patients with between in therapy timings.

Parameters	Therapy timings		Test statistic	
	T1	T2	*t*	*p*-value*
	Mean ± SD			
MNAT-malnutrition risk category	6.05 ± 3.71	20.48 ± 5.73	13.405	<0.001*
WHOQL-OLD	38.63 ± 7.05	73.07 ± 4.82	37.706	

*Paired T: Test, *p*-value.**T1-T2: Therapy Timings (T1: Pre therapy-T2: Post therapy), ***MNAT: Mini Nutrition Assessment Test, ****World Health Organization Quality of Life Scale Elderly Module Turkish Version. MNAT results showed a significant statistical increase after the therapy in our study. In the WHOQOL-OLD quality of life evaluation of our study, the values of 4 parameters, including sensory, autonomy, past, present, and future activities, and social participation, increased gradually toward the T2 (post-therapy) stage, and this increase was statistically significant between the stages (Table 2).

**Figure 5:**
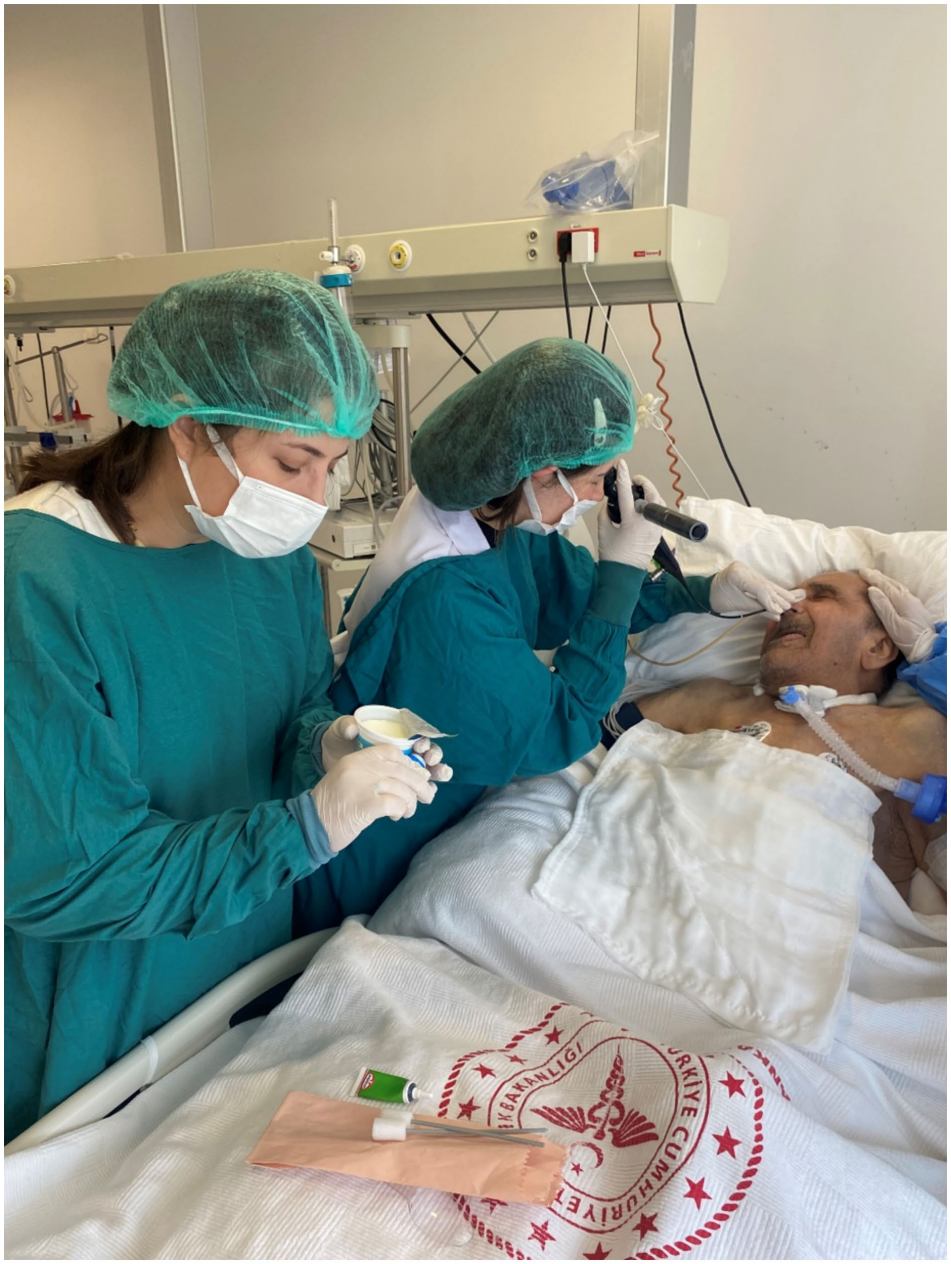
Videofluoroscopic Swallowing Evaluation with older patients hospitalized in the intensive care unit with the complaint of dysphagia.

The EAT-10 scores of therapy timings in older patients show a statistically significant difference (*z* score − 1,828, *p* = 0.003). When the mean score of the bedside water swallowing assessment test, which was performed to evaluate the swallowing function of the patients, was examined, it was observed that dysphagia, which was high in the first follow-up, decreased and that the swallowing function almost returned to normal in the after-therapy follow-up. This difference was found to be statistically highly significant (*p* < 0.01). Bedside water swallowing test results show a statistically significant difference between therapy timings in older patients (*z* score = −2,301, *p* < 0.001) (Table 3). Analysis of MBSImP scores found significant differences in both the “oral total” (OT) score and the “pharyngeal total” (PT) score. Any ineffective swallowing (MBSImP 16: Pharyngeal Residue score of 2 or higher), safe and ineffective swallowing, or any unsafe swallowing (PAS: 3 or higher) In the modified barium swallow study (MBSS), participants confirmed to have difficulties with safe and/or efficient swallowing were identified. In MBSImP, Component 6, the onset of pharyngeal swallowing, T1 (median = 3), T2 (median = 1), Component 8, laryngeal elevation, T1 (median = 1), T2 (median = 0), Component 16, pharyngeal residue, T1 (median = 2), T2 (median = 1). The onset of pharyngeal swallowing, laryngeal elevation, and significant improvement in pharyngeal residual components were observed. Significant improvements in swallowing physiology were represented by improved oral and pharyngeal composite scores of the MBSImP (Table 4). The patient and his/her family are informed about the procedure beforehand. When we look at the results of the interpretation of the VFSS recordings after swallowing therapy was applied, better triggering of pharyngeal swallowing and swallowing reflex was observed in all of the individuals participating in the study. Significant improvement was observed in the pharyngeal residual components. Laryngeal elevation was observed to be better. As a result, it was seen that the patients approached the normal swallowing physiology when compared to the pre-therapy. In our study, when comparing the intertemporal WHOQOL-OLD raw scores, it was 73.07 ± 4.82 at T2 time and 38.63 ± 7.05 at T1 time (Figure 5).

**Table 3:** Comparison of the Eating Assessment Tool (EAT-10) and Bedside Water Swallowing Test (BWST) scores of therapy timings in older patients.

Parameters	Therapy timings		Test statistic	
	T1	T2	*z*-score	*P*-value*
	Median ± SD			
EAT-10 ≥ 3 (dysphagia)	11.52 ± 8.04	4.19 ± 1.01	−1, 828	0.003
BWST Score: Dysphagia (a score of 3–6), a score of 0–2 is believed to have normal swallowing function	3.69 ± 0.81	1.22 ± 0.64	−2, 301	<0.001*

*Wilcoxon testi; **M, Median, SD, Standard deviation, *z* score, *p*-value; ***T1-T2: Therapy Timings (T1:Pre therapy- T2:Post therapy); ****EAT-10, Eat Assesment Test, BWST, Bedside Water Swallowing Test. The EAT-10 scores of therapy timings in older patients with OD show a statistically significant difference (*z* score = −1,828, *p* = 0.003). When the mean score of the bedside water swallowing assessment test, which was performed to evaluate the swallowing function of the patients, was examined, it was observed that dysphagia, which was high in the first follow-up, decreased and that the swallowing function almost returned to normal in after therapy follow-up, and this difference was found to be statistically highly significant (*p* < 0.01) Bedside water swallowing test results show a statistically significant difference between therapy timings in older patients (*z* score = −2,301, *p* < 0.001) (Table 3).

**Table 4:** Comparison of swallowing disorder and physiology before and after therapy.

MBSS	PAS Median ± SD		MBSSImP (Oral phase) Median ± SD		MBSSImP (Pharyngeal phase) Median ± SD	
	T1	T2	T1	T2	T1	T2
Mean	6	3	4	4	9	6
Min	4	3	2	1	6	2
Max	7	6	9	6	14	10
*Z*	−0.39		−2.112		−1.301	
*P*	**0.008**		**0.028**		**0.020**	

*Wilcoxon test, M, Median, SD, Standard deviation, z-scoree, *p*-value. **PAS, Aspiration and Penetration Scale, MPSImp, Modified Barium Swallowing Impairment Profile, MBSS, Modified Barium Swallowing Study. Analysis of MBSImP scores found significant differences in both the “oral total” (OT) score and the “pharyngeal total” (PT) score. Any ineffective swallowing (MBSImP 16: Pharyngeal Residue score of 2 or higher), safe and ineffective swallowing, and any unsafe swallowing (PAS: 3 or higher) In the Modified barium study (MBSS) participants who were confirmed to have difficulty in safety and/or efficiency swallowing were identified. In MBSImP, Component 6, the onset of pharyngeal swallowing, T1 (median = 3), T2 (median = 1), Component 8, laryngeal elevation, T1 (median = 1), T2 (median = 0), Component 16, pharyngeal residue, T1 (median = 2), T2 (median = 1). The onset of pharyngeal swallowing, laryngeal elevation, and significant improvement in pharyngeal residual components were observed. Significant improvements in swallowing physiology were represented by improved oral and pharyngeal composite scores of the MBSImP (Table 4).

Patients with OD further presented a significant decrease in protein, cholesterol levels, and actual body weight before therapy. Handgrip strength was measured as a marker of muscle function. The mean mid-upper arm circumference was 14.03 ± 2.34 cm in patients with malnutrition in the pre-therapy period and 17.48 ± 4.83 cm in patients without malnutrition in the post-therapy stage (*p* < 0.001). When the patients were evaluated according to the Waterlow classification, all patients had normal nutritional status after therapy, and among 25 patients with malnutrition in the pretherapy stage, 22 (88%) had acute, 2 (8%) had chronic, and 1 (4%) had acute malnutrition on a chronic basis. All patients presented high rates of oropharyngeal residue at thin liquids and pudding viscosity with spoon-thick viscosity (MNA ≤ 17) before therapy. Before therapy, impaired nutritional status (MNA ≤ 17) increases the prevalence of patients who need thickeners to maintain a safe swallow and patients with oropharyngeal residue. Safety of swallow was similarly and severely impaired in all patients with (MNA ≤ 17) who presented even lower tongue propulsion forces. Among the patients currently in ICUs who did not use the enteral route and who were in caloric and protein deficit before therapy but who were alive at 12 weeks post-therapy, all achieved satisfactory caloric and protein intake using the oral route (Table 5).

**Table 5:** Nutritional characteristics of older patients with therapy times.

	Before therapy (First week)	After Therapy (Twelfth week)	
Actual body weight	62.15 ± 13.52	78.62 ± 3.21	***p* < 0.05**
%Weight loss	5.57 ± 7.13	0.26 ± 0.65	
Time of weight loss (months)	4.21 ± 1.65	0.487 ± 2.178	
Protein (g/dL)	6.08 ± 0.122	8.0 ± 0.25	
Cholesterol (mg/dL)	152.26 ± 8.24	198 ± 7.46	
Oropharyngeal residue	Thin liquids and pudding viscosity with spoon-thick viscosity (% 100)	0	
Calorie intake ≥80% *N* (%)*	0	25 (%100)	
Protein intake ≥80% *N* (%)*	0	25 (%100)	
The mean mid-upper arm circumference (MUAC)	14.03 ± 2.34 cm	17.48 ± 4.83 cm	
Malnutrition degree nutritional status *(the Gomez classification)*	(Malnourished) Moderate–Severe	Normal	
The rate of malnutrition *(the Waterlow classification)*	Chronic+ acute	Normal	

* Percentage calculated on those still alive in ICU on week 12 (N = 25).

There were significant associations between WHOQOL-OLD scores and the MNA. Clear differences were evident between malnourished and well-nourished patients (on the MNA), those with low and normal arm muscle circumference, and those with good and poor physical function. Regression analysis showed that nutritional scores and functional status contributed independently to the prediction of WHOQOL-OLD (Table 6).

**Table 6:** Spearman’s correlation coefficient for quality of life scores with nutritional variables.

WHOOQL –OLD	
MNAT score	
*R*	0.21
*p-*value	**0.016**
The mean MUAC score	
*R*	0.32
*p*-value	**0.001**

## Discussion

The natural processes of aging and neurogenic diseases have been related to an impairment of neural swallow response that results in delayed laryngeal vestibule closure, oropharyngeal residue, and weak tongue propulsion with the presence of penetrations and aspirations (32). The prevalence of OD among the older population is very high: 23% of older people living in the community, 55% of older people admitted to a hospital with an acute condition, and 40–51% of older people living in a nursing home (32, 33). In frail older patients, 50% of cases of pneumonia come from tracheobronchial aspiration caused by reduced safety of swallowing, presenting a one-year mortality rate of up to 55.4% (34, 35).

Malnutrition risk is linked to a poorer WHOQOL-OLD in older patients on admission to ICUs. Functional status and eating-related factors significantly affect WHOQOL-OLD scores in this patient group. These findings reinforce the role of nutrition as a priority with respect to achieving improvements in WHOQOL-OLD. Studies have shown a direct association between poor nutritional status and worse QoL in some patient groups. Some work has also been reported previously investigating the effect of oral nutritional supplements on QoL in older people (36, 37).

Although studies have shown that dysphagia is primarily found in older patients, the onset time of dysphagia is unknown, and dysphagia may occur in the early period of older patients in relation to functional changes in the cortical swallowing network (38). Oropharyngeal swallowing abnormalities involving aspiration have been found at a rate as high as 45%, and aspiration pneumonia constitutes approximately 70% of the causes of death in older patients (39). Dysphagia has many consequences in patients with AD, including decreased appetite, weight loss, dehydration, malnutrition, functional decline, decreased quality of life with fear of eating and drinking, recurrent lung infections, aspiration, and pneumonia (39). Involuntary weight loss may develop because of both dysphagia and cognitive deterioration. In a previous study, the risk of aspiration was 48% in a patient group with cognitive impairment (40). As a result of malnutrition caused by swallowing difficulties, the immune system may weaken and delay wound healing. The decrease in function leads to increases in morbidity rates, and all these symptoms negatively affect the quality of life. Therefore, Early detection of dysphagia is crucial in older patients (40).

Furthermore, BWST results indicated a statistically significant decrease in dysphagia after swallowing therapy. In the initial diagnosis of dysphagia made with speech and language therapy in the fluoroscopy room of the Radiology Department of Ankara City Hospital, the pharyngeal residue was observed in 71.8% of the patients, penetration in 22.71% of them, and aspiration in 6.5% in the evaluation after VFSS extraction. The mortality rate caused by pneumonia in older patients is high, especially compared to that in patients without dementia (41, 42). Therefore, it is necessary to detect the signs of dysphagia in advance.

Older patients with OD are often associated with oral and pharyngeal impairment, and VFSS is the gold standard and important for its diagnosis and treatment (43–46). The IDDSI framework and testing techniques can be used to ensure the suitability of any food or liquid (18). Previous studies have assessed swallowing function in hospitalized older patients using VFSS (47, 48). In addition, the oral function of institutionalized older patients has been assessed using simple measures, such as interviews with caregivers, questionnaires, and observation of the oral cavity (49, 50). In other studies, oral function evaluation included multiple swallowing assessments for mild cognitive impairment (MCI) and mild dementia (51, 52) or caregivers’ assessments of feeding and swallowing functions (53).

Logemann et al. (54) found that the prevalence of aspiration was 39% in patients with Parkinson’s disease. They evaluated the swallowing functions of older patients using FEES and detected moderate swallowing disorders in 12 of 21 patients on instrumental examination, although only 4 of 21 patients reported dysphagia. Swallowing and eating may be observed in older patients due to motor involvement; low thrust may result from reduced tongue movement. Prolongation of oral and pharyngeal transit, decreased hyolaryngeal elevation, delayed closure of the laryngeal vestibule, and decreased opening of the upper oesophageal sphincter may occur. There may be a decrease in the ability to clear the bolus due to the decreased force of contraction of the tongue, pharynx, and larynx (55). As a result, swallowing may result in oral or pharyngeal residues, and penetration and/or aspiration may be observed in patients (55). In this study, BWST, clinical swallowing, and instrumental swallowing evaluations were performed. Swallowing therapy was administered for 12 weeks (14−16 sessions). Previous studies have indicated that the water swallowing test predicts dysphagia. These studies evaluated the finding of cough after the water swallowing test in the presence of dysphagia in 30−45% of stroke patients (56). There is also a study in which BWST, pulse oximetry, and O_2_ saturation evaluation are performed together. This study observed that older patients whose swallowing scores were calculated through the bedside swallowing test had severe dysphagia, but there was a decrease following swallowing therapy.

Dinsever Eliküçük et al.’s study (57) compares the effectiveness of short-term and long-term swallowing therapy in older patients with VFSS. The researchers found that the penetration/aspiration score was higher in the short-term treatment group than in the long-term group. They proved that long-term swallowing therapy programs are more effective than short-term therapy in older patients with swallowing difficulties.

Standard swallowing tests are difficult for older patients (55, 56). Evidence on clinical evaluation, intervention, and medical treatment of dysphagia in older patients is still limited (54–56). Very few studies have evaluated swallowing function in this patient group (58, 59). Although oropharyngeal swallowing abnormalities, including aspiration, are found at a rate of up to 45%, aspiration is more common in older patients (59). Aspiration pneumonia constitutes approximately 70% of the causes of death in older patients (43, 58, 59).

Many studies have used MNAT as the gold standard test for the screening and evaluation of malnutrition in older patients (46). Therefore, the MNAT was used in our study. Following therapy, the MNAT results of the patients increased significantly, and their nutritional status improved. In some studies, as in our study, the relationship between MNAT and dysphagia was also examined. In a previous study in which they looked at therapeutic activities in older patients with dysphagia, Tang et al. (43) evaluated 103 older patients, 53 with dysphagia and 50 with normal swallowing, with a mean age of 72.5 years. They used VFSS, MNAT, and sMMT in their study. They found a mean of MNAT 9.3 in patients with AD with an SMMSE of 13 ± 4.2. In a study of older patients with dysphagia and pneumonia, Cabre et al. (44) estimated that the prevalence and risk of malnutrition were 36.8 and 55.3%, respectively, in those with dysphagia compared to those without dysphagia. Saleedaeng et al. (45) found that 268 older patients were admitted to the hospital for various reasons and a statistical difference between the meal patterns among the older patient group with dysphagia and the normal-swallowing older patients group at the 0.05 level. According to this study, dysphagia was associated with the nutritional status of older patients and dietary patterns. Suh et al. (46) found pharyngeal residue in 80%, penetration in 73.3%, and aspiration in 13.3% of older patients, whose mean age was 73.93 and median SMMSE was 9.15. In our study, pharyngeal residue was observed in 71.8, penetration in 22.71, and aspiration in 6.5 of all cases. Residues are mostly observed in the vallecula of older patients. Although residual vallecula is observed only in older patients, 50% of those with residuals only in the pyriform sinus are older patients with clinical bedside evaluation, and the degree of bolus misdirection was overestimated in 15 patients with minor aspiration and underestimated in 10 with major aspiration (46).

The severity of the disease has been found to be more effective than dysphagia in the deterioration of quality of life. A total of 91.3% of these older patients were malnourished. Impairment of swallowing function negatively affects the nutritional status of individuals and increases the risk of malnutrition. Information on how the quality of life of older patients changes over time is limited (44, 46). The increase in the WQOOL-OLD score indicates an increase in quality of life, which was statistically significant after therapy. In the WHOQOL-OLD quality of life evaluation of our study, four parameter values—sensory, autonomy, past, present, and future activities, and social participation—gradually increased after therapy (T2 time), and this increase was statistically significant between intervals. The decrease in the WQOOL-OLD score indicates a decrease in the quality of life, which was statistically significant over time. When intertemporal WQOOL-OLD raw scores were compared, T2 and T1 times were 72.11 ± 4.03 and 47.22 ± 7.18, respectively.

In all cases, SMMSE, daily life activities, quality of life results (*p* < 0.05), swallowing function, and nutritional status (*p* < 0.05) improved significantly after therapy. Considering the EAT-10 median results, this rate was higher in individuals with aspiration penetration than in others. In one of the items of the FOIS scale, 18 of the individuals who took ‘full oral intake without special devices but with specific food restriction’ were given full oral intake before therapy, while 16 continued to use full oral intake, and two of them continued to use a nasogastric tube. Moreover, 12 of those who had full oral intake without restriction were found to be after therapy. The FOIS status showed statistically significant differences based on therapy timing. According to the results of the EAT-10 swallowing screening questionnaire, complaints of dysphagia showed a statistically significant decrease after therapy.

Swallowing screening questionnaires in older patients is compatible with objective evaluation results when information is obtained from their relatives (46). However, receiving information about swallowing complaints from these individuals negatively affected the accuracy of the results. Therefore, we used the scales to ensure our study’s test results were reliable.

Although swallowing function and nutritional status deteriorate as the disease progresses, individuals should be evaluated in terms of malnutrition and dysphagia; early preventive and therapeutic strategies should be developed to prevent conditions such as aspiration pneumonia that may develop in the future, and the patient and their family should be guided. There are very few studies on dysphagia screening in older patients with OD, dementia AD, and so on (24, 25, 54, 60, 61). To discuss the studies that have similar aspects to the latest current study:

Wu et al. (24) conducted a study in which stepwise swallowing training (SST) integrated with the movement of all swallowing organs anticipated swallowing dysfunction in older patients. In their method, they implemented an SST program (including five parts of swallowing organ training: lip movement, facial movement, tongue movement, mandibular movement, and neck movement) with a 4-week follow-up period. They assessed swallowing function using the Water Swallow Test (WTS) and the Standard Swallow Assessment (SSA). They measured the potential for adverse effects of dysphagia on eating performance, daily activity ability, and nutritional status. Data were collected at baseline (T1), 2 weeks (T2, intervention), and 4 weeks (T3, follow-up) with modification. The SST product improved both swallowing function, and the adverse effects of dysphagia persisted (24). Compared to ours, the weakest aspect of their study is that they did not use the gold standard FEES or MBSS test as we did.

Logemann et al. (54) conducted a study using thin liquids containing chintuck or nectar/honey-thickened liquids in individuals with dementia or Parkinson’s disease (PD). They found less compensation in the desire for nectar and honey-thickened liquids.

Espinosa-Val MC et al. (60) performed the oropharyngeal dysphagia (OD), volume-viscosity swallow test, and a geriatric assessment with an older patient. Researchers applied rapid therapeutics to OD patients with products that have modified fluid viscosity, texture, and oral hygiene, and they are being followed up for 18 months. In their discussion, the researchers stated that the new program should be reworked to increase compliance and therapeutic effects in this growing group of older patients with dysphagia.

Shirobe et al. (25) stated that oral function assessments in older patients are important to determine appropriate and practical dietary support plans. However, they emphasized that it can be challenging to cooperate in understanding and evaluating instructions. This study aimed to determine the feasibility of oral function assessments in individuals with AD according to the Functional Assessment Staging of Alzheimer’s Disease (FAST) stages.

They were used to examine the prevalence of participants who were unable to perform oral function evaluations, including oral diadochokinesis (ODK), repeated saliva swallow test (RSST), and modified water swallow test (MWST). The study concluded that oral function assessment in older patients is difficult and that simpler and more practical swallowing function assessments that can be routinely observed and the frailty of aging are needed.

Michel et al. (61) aimed to define the prevalence of OD in community-dwelling older patients using the V-VST, the reference clinical screening test for swallowing opportunities in a population from a geriatric outpatient clinic. OD screening was performed using V-VST during consultation. The detection of cognitive impairment was determined by SMMSE, and OD detection was determined by the Dysphagia Outcome Severity Scale (DOSS). Six geriatric domains were assessed (comorbidities, functional abilities, cognition, nutrition, mood disorders, and frailty). The study results indicated that OD is a very common occurrence in elderly people with dementia living in the community and that V-VST is an easy and well-tolerated screening test in this group and, therefore, should be systematically included in the geriatric assessment of older patients. The study is limited by the fact that the role of V-VST in the treatment of OD has not yet been evaluated.

As a result of our examinations in all studies, swallowing exercises, the fundamental rehabilitation for dysphagia, need more high-quality evidence regarding their implementation in older patients. These exercises can effectively increase the flexibility and coordination of swallowing organs and improve the relevant muscles’ strength. Nonetheless, whether swallowing exercises are effective in older patients is controversial. Therefore, simple and feasible swallowing rehabilitation training should be developed for patients with impaired cognition and attention deficit.

Dysphagia studies and rehabilitation programs in the geriatric group (stroke patients, older patients with intellectual disability, and so on) (62–65); Kang et al. (65) studied a bedside exercise program consisting of oral, pharyngeal, laryngeal, and respiratory exercises in stroke patients. After a performance of 1 h per day for 2 months until the 25th year of the program, the results showed a significant improvement in swallowing performance (oral phase) and depression compared to the control group. Eliküçük et al. (62) reported the positive effect of long-term swallowing therapy on PAS scores in older patients. In their findings, when the post-therapy penetration aspiration scores and pharyngeal residual severity scores of individuals receiving long-term and short-term therapy were compared, the short-term therapy group had a higher score than the long-term group, and a significant difference was found. Twenty-seven (88.4%) patients had pharyngeal phase abnormalities, and 23 (75.3%) had laryngeal penetration/aspiration, and both variables were significantly higher in the short-term group. In the long-term therapy group, EAT-10 scores obtained before therapy were significantly higher than EAT-10 in the third month after therapy. 78% of patients experienced silent aspiration of thin liquid, nectar, and solid consistency before therapy. In their study, a long-term swallowing therapy program was found to be more effective than short-term therapy in geriatric individuals with dysphagia. The similarity between this study and our study is the improvement in PAS and EAT-10 scores after 12 weeks of therapy in older patients.

Interestingly, in this study, although penetration aspiration scores appeared to improve at T2, MBSImP oral and pharyngeal composite scores worsened at T2. We attribute this result to the following circumstances: Abnormal swallowing function in older patients manifested as weak tongue movement and pressure, generally occurring in the oral and pharyngeal stages of swallowing. Hence, individuals might exhibit delayed pharyngeal reflex, reduced pharyngeal muscle strength, and food residue after swallowing due to abnormal swallowing function. Individuals with abnormal swallowing function experience difficulty in forming and pushing the food bolus and, hence, are at high risk of food aspiration (66). In the early stages of older patients, dysphagia is more likely to occur because of oral dysfunction, such as decreased tongue motor and masticatory functions and challenges in the oral phase of swallowing (i.e., the first stage of the swallowing process), such as delayed swallowing reflexes. Although oral phase challenges are associated with longer mealtimes and the risk of malnutrition, they do not pose clinical challenges and are often unnoticed by caregivers (25). The results of our oral component scores also support that; therefore, the evaluation of oral function, including swallowing function, is important in older patients. However, dysphagia in older patients is usually characterized by oral and pharyngeal phase problems, including problems of pharyngeal clearance, upper esophageal sphincter opening, and visible aspiration on VFSS (21, 47–53, 61, 67–77). We attribute the worsening in the oral and pharyngeal component parameters of our study, as seen in these studies, to the fact that each substance was examined separately and handled more specifically.

Previous research showed that in the ICU, underfeeding is very common due to the inability to deliver the required amount of nutrients (26, 27, 78). Repetitive fasting periods, enteral tube complications, and gastrointestinal intolerance are the most frequently reported problems (79).

According to Jackson et al. (80), the first trigger for growth is fat deposition, which is directly proportional to an increase in skinfold thickness and weight gain. There is a known relationship between basal oxygen consumption and weight for age and skinfold thickness. In the present study, the mid-upper arm circumference was lower in patients with malnutrition before therapy. Among the patients currently in ICUs who did not use the enteral route and who were in caloric and protein deficit before therapy but who were alive at the 12-week post-therapy stage with swallowing and nutrition therapy, all achieved satisfactory caloric and protein intake using the oral route.

There is increasing evidence demonstrating the high prevalence of malnutrition in older patients (81, 82). The systematic review (15) suggested that individuals at high risk of malnutrition are more likely to experience poor QoL and that interventions designed to improve nutritional status can also significantly improve QoL in older patients. Systematic reviews exploring the potential benefits of nutrition support have indicated that nutritional supplementation positively affected nutritional outcomes and mortality in older patients (82, 83).

We found significant associations between WHOQOL-OLD scores and the MNAT in older patients in the ICU. Clear differences were evident between malnourished and well-nourished patients (on the MNAT), those with low and normal arm muscle circumference, and those with good and poor physical function. Regression analysis showed that nutritional scores and functional status contributed independently to the prediction of WHOQOL-OLD. The effect on food intake, mobility, and psychological stress/acute disease also had a significant influence.

### Guidelines to assist management of nutrition in older patients

Malnutrition has been associated with increased mortality and complication rates and a longer ICU stay, and its prevalence is higher among the critically ill than among other hospitalized patients (84, 85).

In Isenring et al.’s study, nutrition screening tools used included the MNA-short form Malnutrition Screening Tool (MST), the Malnutrition Universal Screening Tool (MUST), the Simplified Nutritional Assessment Questionnaire, and the anthropometric screens. Corrected arm muscle area and calf circumference have acceptable concurrent validity compared with validated nutrition assessment tools and can be used to triage nutrition care in the long-term-care setting (78). The common malnutrition screening tools and available anthropometric measures are inappropriate in critical illness, mostly due to the significant fluid shifts that occur, preventing accurate assessment of weight and other anthropometric assessments. Many of these available screening tools have not been validated or are unable to be validated in the ICU population. Increasingly, the use of feeding tubes (i.e., PEG tubes) has been challenged by empirical research, which has not supported the rationales provided for this intervention. Over the past 15 years, research has shown that referring to tube feeding for older patients as life-sustaining or life-prolonging is inaccurate. Studies have shown that feeding tubes “provide no protection from aspiration pneumonia and may even increase the risk. Survival rates are similar between those with older patients who have feeding tubes and those who do not (67–69, 74, 75).

Lahey et al. (67) reported that researchers believe that older patients have significantly reduced nutrition due to the associated muscle recovery and immobility. Researchers and students have advocated for careful hand-feeding protocols and nutritional diets to provide nutrition to older patients. They emphasized that hand-feeding provides the added benefit of allowing older patients with sensory deprivation to experience both deprivation and the touch of a caregiver. They have also increasingly recommended against the use of feeding tubes for older patients. In our study, a similar aspect is that we support the hand-feeding method to avoid using PEGs. At the same time, we continued oral feeding in all our patients by adding diet modifications to our holistic therapy program.

Robison et al. (70) found dysphagia interviews in 5.4% (*n* = 47,574) of older patients who screened for dysphagia and had oral dysphagia (*n* = 21,438, 2.4%), pharyngeal dysphagia (*n* = 24,257, 2.7%), and general swallowing complaints/pain (*n* = 14,928, 1.7%). Overall, PEG feeding tubes were placed in 3529 patients (11.2%) with prehospital dysphagia, compared with 27,893 patients (88.8%) without prehospital dysphagia, according to a Minimum Data Set (MDS) 3.0 assessment. They found that the risk of feeding facility placement increased with the number of dysphagia items not present on the prehospital MDS (6 vs. 0 dysphagia variables).

Kumagai et al.’s (71) study with older patients concluded that PEG tube feeding does not appear to support medical care at home.

Nakanishi and Hattori’s (72) results question whether PEG tube feeding can improve QOL among older patients. They noted that national health policy was not informed to explore an intervention that would help patients, their caregivers, and practitioners make decisions about feeding options. Additionally, the importance of the role of SLT in PEG tube feeding was included in Nakanishi and Hattori’s study.

Despite the lack of evidence that enteral feeding tubes benefit older patients, and often contrary to the wishes of patients and family, older patients who have difficulty swallowing or reduced food intake often receive feeding tubes when hospitalized for an acute illness. Monteleoni and Clark (73) found that the number of feeding tubes placed in all older patients receiving percutaneous endoscopic gastrostomy or jejunostomy tubes was significantly reduced after the interventions. Goldberg and Altman (76) emphasized the importance of training caregivers and clinicians in the management of dysphagia in gastrostomy tube placement in advanced dementia with dysphagia. They said that caregivers, and often health workers, do not have all the information before making a decision. Therefore, when deciding whether and when to insert a PEG, the data should be reviewed by healthcare staff and family members. Caregivers need to be educated about the quality of life of full per-oral nutrition normal diet (PO) feeding, which can be interpreted as continuing to feed patients orally, following the safest diet, and taking strict aspiration precautions. Throughout the therapy, caregivers had the chance to observe and practice with SLPs at every stage in our dysphagia management and feed patients orally, following the safest diet and taking strict aspiration precautions. We have not had any cases where PEG use was felt during therapy. We believe that we owe this situation to the fact that our therapy program is based on a holistic and systematic infrastructure that meets every need.

## Limitations

The lack of a control group is a limitation of the study. It was very difficult to recruit a second group in a very difficult group – in this group with older patients and under intensive care conditions. We aimed to determine the optimal number of therapy sessions by determining the study group according to the inclusion criteria. There were very few previous swallowing therapy studies in this difficult older group. Therefore, a control group was not included. The therapy journey of an older patient required a very meticulous therapy journey adventure, and the multidisciplinary team had to be present at every stage under intensive care conditions. For these reasons, our study was conducted in only one study group. In other diagnostic groups, hospitalized in intensive care, routine nursing services and dysphagia evaluations are performed in individuals with dysphagia risk.

Second, some participants were unable to complete the questionnaires themselves due to poor eyesight, disabilities that made writing difficult, or they simply felt too ill. They did not wish to complete them without help. In this situation, the questionnaires were read aloud and then completed based on the participants’ responses. Although care was taken not to prompt replies or to suggest which response was most appropriate, this may have affected how they answered. There is also some potential for bias because WHOQL-OLD measures were not administered blindly to nutritional assessments. Although the nutritional data were collected simultaneously as the WHOOQL-OLD questionnaires were administered, these questionnaires were scored separately.

## Conclusion

Bedside tests and clinical and instrumental swallowing assessments provide fast and practical results for dysphagia evaluation in the early stages, especially in older patients. In older patients hospitalized in the intensive care unit, cognitive and behavioral disorders are emphasized, and the problem of dysphagia is generally ignored. Very few studies have evaluated and treated dysphagia in older patients.

Therefore, more studies on the evaluation and treatment of dysphagia in this patient group are needed. Aspiration pneumonia is the most common cause of death, particularly in older patients. Therefore, older patients should be inspected in detail in the initial stage in terms of dysphagia, and exercise-based swallowing therapy (dysphagia treatment) should be applied with the necessary preventive measures. Diet modification support should be provided using oral feeding methods or other alternative strategies. Furthermore, the families and caregivers of patients should be informed about dysphagia and aspiration symptoms. With appropriate swallowing therapy programs, it is possible to shorten hospital stays, increase functional status, and reduce complications and mortality. The use of PEG tubes in older patients did not show benefits with regard to survival, improvement in QOL, or reduction in aspiration pneumonia. There is a significant lack of evidence supporting the evaluation and management of dysphagia in older patients.

The results reported in the present study show that malnutrition risk is linked to a poorer QoL in older patients on admission to ICUs. Statistical analyses revealed the dominant effects of functional status and eating-related factors on QoL in this group. The present findings reinforce the role of nutrition as a priority for improving patients’ perceptions of QoL. Further studies are required to investigate and identify the interventions that improve QoL in older patients. To establish whether nutritional intervention is effective in enhancing.

QoL in this vulnerable group requires more studies with better research designs.

## Data Availability

The datasets presented in this study can be found in online repositories. The names of the repository/repositories and accession number(s) can be found in the article/supplementary material.
